# Study of cognitive impairment in depression

**DOI:** 10.1192/j.eurpsy.2021.878

**Published:** 2021-08-13

**Authors:** R. Sharma, J. Khattri, P. Thapa

**Affiliations:** 1 Psychiatry, National medical college, kathmandu, Nepal; 2 Psychiatry, Manipal College of Medical Sciences, Pokhara, Nepal

**Keywords:** Depression, cognitive functions

## Abstract

**Introduction:**

Cognitive impairment is frequently observed in patients suffering from depression. Cognitive dysfunction play a critical role in increasing the individual’s vulnerability for the first onset, maintenance and future recurrence of depressive episodes.

**Objectives:**

The objectiv was to assess the cognitive impairment in patient with depressive episode.

**Methods:**

A cross sectional, hospital based study was conducted among 100 patients with depressive episodes diagnosed by International Classification of Diseases - 10 visiting outpatient and inpatient in Department of Psychiatry of Manipal Teaching Hospital, Pokhara, Nepal. The subjects were interviewed with Beck’s depression inventory, Perceived deficient questionnaire, Frontal assessment battery, Trail making test A and B and Forward and Backward Digit Span test. For the assessment of correlates, regression analyses were done using SPSS v 20.0.

**Results:**

The mean age of the participants was 32.47 years (SD±12.25), majority were female, married, Hindu and from urban population. Higher number of respondent were student. Most of them were educated till intermediate level and belonged to middle socioeconomic class family. Different domain of cognitive function according to severity of depression was found to be statistically significant (p<0.05). This study also found that age, sex, education, medication use and Becks depression inventory score predicted the cognitive function.

**Conclusions:**

Cognitive impairment is not ucommon among patient with depressive episodes. The impairment is not only seen in severe cases but also in mild to moderate cases. The assessment of cognitive deficits should be the regular part of the assessment in depressive patients.

